# Potential Point-of-Care Microfluidic Devices to Diagnose Iron Deficiency Anemia

**DOI:** 10.3390/s18082625

**Published:** 2018-08-10

**Authors:** Boon Kar Yap, Siti Nur’Arifah M.Soair, Noor Azrina Talik, Wai Feng Lim, Lai Mei I

**Affiliations:** 1 Electronics and Communication Department, College of Engineering, Universiti Tenaga Nasional, KM-7 Jalan Uniten-Ikram, 43000 Kajang, Selangor, Malaysia; sitinurarifah.msoair@gmail.com (S.N.M.); azrina_talik@hotmail.com (N.A.T.); 2 Institute of Power Electronics (IPE), College of Engineering, Universiti Tenaga Nasional, KM-7 Jalan Uniten-Ikram, 43000 Kajang, Selangor, Malaysia; 3 Integrative Pharmacogenomics Institute (iPROMISE), Universiti Teknologi MARA Selangor, Puncak Alam Campus, 42300 Bandar Puncak Alam, Selangor, Malaysia; limwaifeng85@gmail.com; 4 Department of Pathology, Faculty of Medicine and Health Sciences, Universiti Putra Malaysia, 43400 UPM Serdang, Selangor, Malaysia; Imi@upm.edu.my

**Keywords:** microfluidic, iron deficiency anemia, point-of-care, all-in-one

## Abstract

Over the past 20 years, rapid technological advancement in the field of microfluidics has produced a wide array of microfluidic point-of-care (POC) diagnostic devices for the healthcare industry. However, potential microfluidic applications in the field of nutrition, specifically to diagnose iron deficiency anemia (IDA) detection, remain scarce. Iron deficiency anemia is the most common form of anemia, which affects billions of people globally, especially the elderly, women, and children. This review comprehensively analyzes the current diagnosis technologies that address anemia-related IDA-POC microfluidic devices in the future. This review briefly highlights various microfluidics devices that have the potential to detect IDA and discusses some commercially available devices for blood plasma separation mechanisms. Reagent deposition and integration into microfluidic devices are also explored. Finally, we discuss the challenges of insights into potential portable microfluidic systems, especially for remote IDA detection.

## 1. Introduction

Iron deficiency anemia (IDA) is a nutritional disorder and is the most common anemia in the world, affecting billions of people globally. In 2011, IDA accounted for 50% of the anemia cases reported worldwide [1]. Iron deficiency anemia is caused by an iron imbalance, whereby the iron absorbed cannot compensate for the iron lost by the body, and the resulting lack of iron, impair hemoglobin synthesis, leading to fatigue, low productivity, and pallor [2,3]. Iron deficiency anemia is frequently observed in infants, toddlers, and childbearing women [4,5]. In addition to decreased productivity and premature death, IDA can also have a long-term effect on the patient’s neurodevelopment [3,6,7]. However, the general symptoms of IDA are similar to those of other types of anemia. Without proper investigations, even the initial screening of thalassemia, a hemoglobin disorder, can be misdiagnosed as IDA. In addition, the catalysts used in the diagnostic processes of iron supplements may contaminate patients and even lead to death [8], which has led to research into ways to determine and specify the amount of metals in iron supplements for IDA treatment [9]. Incorrect diagnosis could increase the severity of the disorder. Thus, further investigations must be devoted to develop accurate diagnosis methods. One method that ensures the correct diagnosis and treatment of IDA is to assess the quantity of iron stored in the body. In the gold standard assessment method, bone marrow examination is conducted. However, this biopsy is well known to be invasive and risky. 

Extensive research has been conducted to invent and improve new and existing microfluidic point-of-care (POC) diagnostic devices. One of the prominent POC-based technology developed for the medical industry uses microfluidics [10,11,12,13,14,15,16,17]. Today, however, few microfluidic POC diagnosis tests are available in the market for detecting various diseases such as human immunodeficiency virus (HIV) [18], malaria [19,20], dengue virus [21], and rare cell mutations [22]. These tests are simple, economical and offer a rapid detection rate.

Despite these developments, a specific integrated POC-based microfluidics diagnosis device (i.e., sample collection, pre-treatment, on-chip filtration, reagent mixing, signal detection and readout) [23] for IDA remains unavailable. In conjunction with the campaign of the World Health Organization to tackle micronutrient deficiencies in developing countries [24], this review discusses the challenges faced for implementing a low-cost IDA-POC diagnosis based on microfluidic technologies. This paper, comprehensively analyzes the microfluidic technologies currently available for anemia-related disease. First, diagnosing tests for IDA is presented. Next, potential microfluidic devices for IDA detection are briefly highlighted, following which commercially available blood plasma separation mechanisms are discussed, and reagent deposition and integration into microfluidic devices is examined. Finally, we discuss the challenges and present insights into the possible future of portable microfluidic systems, especially for remote IDA detection. 

## 2. Diagnosing Iron Deficiency Anemia

In order for a patient to be diagnosed as anemic that is iron deficient, the patient must be anemic and have evidence from laboratory that shows iron deficiency. Currently, the most common screening technique for IDA and iron overload is the detection of the serum ferritin level. Patients with less than 10 mcg per dl (100 mcg per L) serum ferritin indicate the highest likelihood to suffer from iron deficiency anemia. Other biochemical markers may be used to assess the quantity of iron stored in the body, including serum iron concentration, total iron-binding capacity, transferrin saturation, serum transferrin receptor, as well as zinc protoporphyrin [3]. The latest approach of detecting IDA is by observing the level of serum transferrin receptor. Table 1 show that patients with IDA will have a high level of total iron-binding capacity and low level of transferrin saturation. This is because the availability of amount of iron to bind to the iron-carrying protein, transferrin has been reduced. 

However, each biochemical marker has its individual advantages and disadvantages. For example, ferritin concentrations are affected by inflammation, tissue damage, neoplastic disease, or increased metabolism. The instruments currently available for IDA diagnostic tests are bulky and costly (e.g., biochemistry auto-analyzers or red cell auto-analyzers). The costs of these machines can be prohibitive, especially in rural and underdeveloped regions. In addition, skilled personnel are required to draw the blood samples for the diagnosis, and transporting this samples to the relevant centers increase costs. Furthermore, such samples are prone to physical damages and to biochemical changes in blood. Thus, a user-friendly, economical, rapid, and accurate point-of-care (POC) device is urgently required to address IDA, particularly in poor developed country, and developing countries.

## 3. State-of-the-Art Microfluidic Devices for Iron Deficiency Anemia Detection

**Figure 1:**
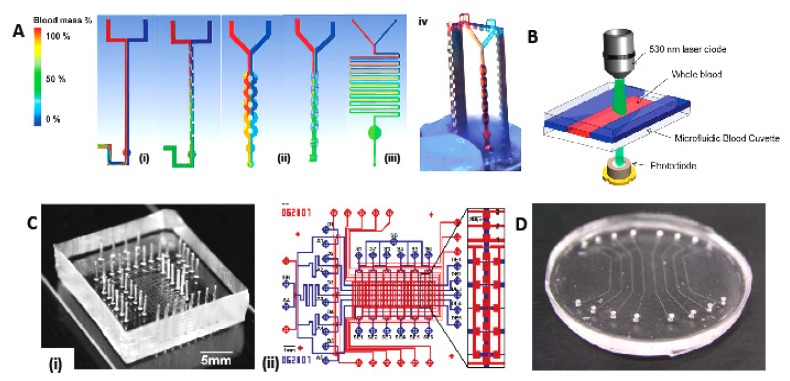
(**A**) Design of three sophisticated POC system iPOC^3D^ microfluidic mixers simulated by 3D computational fluid dynamics: (**i**) Split-and-recombine (SAR) mixing, (**ii**) ring-shaped channel, and (**iii**) serpentine channel. Red flow is blood mixed with an aqueous solution (blue); green indicates complete mixing. (**iv**) Image of 3D printed, 3D-structured micro-mixer showing efficient mixing within 1 s using colored dye solutions [25]. (**B**) Setup for measuring hemoglobin concentration [26]. (**C**) Microfluidic immunoassay chip from Kartalov’s group. (**i**) The experiment used this 60-chamber polydimethylsiloxane (PDMS) chip bound to a 1-inch-wide epoxide slide. (**ii**) Architectural diagram of the test column [30]. (**D**) Eight-channel PDMS microfluidic device for ferritin immunoassay detection demonstrated by Schrott et al. [32].

This section reviews the latest microfluidic designs that have the potential to further improve IDA-POC screening. Recently, Plevniak’s group demonstrated a sophisticated POC system called iPOC^3D^ to diagnose anemia [25]. This device can be integrated with smartphones for rapid testing via colorimetric measurement. The outstanding characteristic of this device is the three-dimensional (3D) printable channels for fast self-mixing of blood and reagent (within 1 s), where merely 5 μL blood assay is required as sample to measure the hemoglobin. Figure 1 shows several self-mixed designs of iPOC^3D^ by 3D computational fluid dynamics. These designs account for the force balance between surface tension and viscous forces and ignore the gravitational force because of the low bond number (≤1) in the micro-scale tube. The ring-shaped channel [Figure 1A (iii)] is adapted for the final testing by combining the split and recombination (SAR) mixing [Figure 1A (i)] and serpentine design [Figure 1A (ii)]. Rapid self-mixing is achieved through the high rate of fluid-flow in the ring channels.

Another state-of-the-art technology for measuring hemoglobin using a microfluidic system was proposed by Kim et al. [26], which involves, a novel single-side-wall air-bubble-free microfluidic cuvette. Figure 1B shows the setup for measuring hemoglobin concentration for this device. A 105 μm-deep microfluidic chip utilizes capillary force action to make the sample flow. A channel of appropriate height needs to be properly chosen because the capillary force increases as the channel height decreases [27]. The experiment of Kim et al. [26] uses the cyanmethemoglobin method wherein the blood sample is mixed with Drabkin’s solution (containing ferricyanide and cyanide) to avoid blood-cell aggregation. The maximum absorbance of the subsequent chemical reaction is at 540 nm. A 530-nm laser diode serves to measure the sample absorbance. 

These inventions help diagnose anemia. However, for IDA detection, measuring hemoglobin level alone is not sufficient. For instance, red cell distribution width of less than 15% in hemoglobin may indicate IDA or anemia of mixed origin [28]. Misdiagnosis for IDA may occur because diagnosing a malnutrition disease such as IDA merely from anthropometric indicators is not straightforward unless they are severe [13]. In contrast, these inventions could be further improved by integrating on-chip filtration for blood-plasma separation with an automated sample processing and readout system to enable IDA detection from a single-biomarker. 

To date, studies performed using IDA biomarkers have concentrated only on developing immunoassays for microfluidic scales. For instance, Kartalov et al. demonstrated a-proof-of-principle microfluidic device that uses multiple antigens, including serum ferritin [Figure 1C] [29,30]. Arrays of micro-mechanical valves were integrated to control the pressure and drive the flow of the multiplex antigen and reagent (as in an enzyme-linked immunosorbent assay). Their first work used a simple buffer solution for detection [29], which was then extended to complex biological samples in their second work. They used the device to analyze several protein analytes in human serum such as ferritin, thyroglobulin, C-reactive protein (CRP) and also prostate-specific antigen. The device measured human serum ferritin down to 250 pM [30]. In addition to that, it uses in-chip calibration by sample spiking with a known concentration of antigen. This low-cost and efficient innovation could provide accurate IDA diagnosis if integrated with the inflammation and infection analyte, C-reactive protein [31]. For signal processing to quantify the analyte, the device uses a laboratory size fluorescence microscope. Another ferritin immunoassay device that uses microfluidic approach was proposed by Schrott et al. [32,33] who demonstrated eight-channel poly(dimethylsiloxane) (PDMS)–based ferritin immunoassay [Figure 1D]. The ferritin immunoassay was performed using proteins such as rIgG [Rockland 200-401-090, anti-ferritin (human spleen) rabbit IgG], antigen ferritin, secondary anti-body b-rIgG [Rockland 200-406-090, biotin-conjugated anti-ferritin (human spleen) rabbit IgG], and FITC-avidin. These proteins were immobilized in micro-channels prior to signal detecting the fluorescence signal via an external fluorescence detection system. The device was tested with a sensitivity better than 6.7 pM using mouse antibodies [33]. Unfortunately, similar to the works explained hitherto, the absence of on-chip filtration and a signal-readout system limit the potential of this device for IDA-POC detection. 

### 3.1. Filtration and Mixing Micro-Device for Blood-Plasma Separation

To date, most of the samples used for IDA diagnosis have been serum samples. However, serum samples are procured from clotted whole blood, which requires a certain amount of time. Thus, plasma samples, which have the same composition as serum samples without the clotting-related factors, should be used for rapid diagnosis. 

#### 3.1.1. State-of-the-Art Blood Plasma Separation and Filtration

Conventionally, blood can be separated into three fractions through centrifugation, but this requires intensive labor and is not easily accessible. Microfluidic technology possesses a significant potential to address these issues. The Navier-Stokes equation is normally adapted for incompressible fluid dynamics, which is the relevant regime in microfluidics platforms to control fluid flow. The equation is:

(1) $$\rho \;\left[\;\frac{du}{dt}+\left(u\cdot \nabla \right)u\;\right]=-\nabla P+\eta \nabla^{2}\mu$$

where **u** is the fluid velocity, *P* is the pressure, and η is the fluid velocity. The equation shows that on the right side, the force per unit volume is due to a pressure gradient (−∇P) and viscosity (η∇2μ). The mass per unit volume ρ, times the acceleration of fluid is expressed in terms of the convective derivative in a Eulerian representation on the left side. Additional body forces acting on the fluid is summed to the right-hand side [34]. 

**Figure 2:**
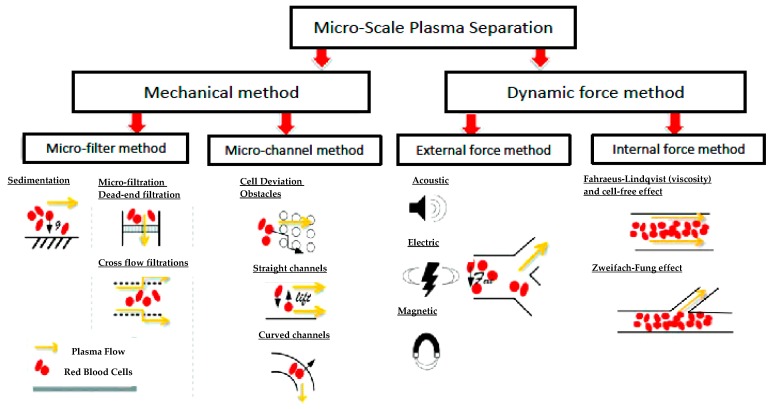
Technological solutions for micro-scale plasma separation. The image is adapted and modified from Reference. [45]–Published by The Royal Society of Chemistry.

There are two major classifications for the fluid-flow method: the mechanical method and dynamic force separation. The mechanical method can be further be divided into two approaches based on the devices used: microfilter based devices [35] and microchannel based devices [36,37]. The dynamic force separation method can also be divided into two approaches wherein; (i) an external force such as magnetic, optical, electrical, acoustic, or thermal force is used and (ii) a centrifuge and internal force using hydrodynamic characteristic such as viscous force, shear stress, effects due to device geometry [38]. Figure 2 illustrates the methods demonstrated to date for blood-plasma separation. To ensure that a device can be used in all diverse environments, including remote regions, plasma separation devices should be autonomous and not require an external power source. In-depth discussions about fluid-flow methods are available in other review papers [23,34,39,40,41,42,43,44]. 

This section discusses the commercially available devices that use blood-separation technology. In the future, the know-how-technologies implemented in such commercialized devices can be applied to IDA-POC microfluidic devices. The most important parts of a low-cost POC system are the sample collector and processing system. Most commercialized devices come with a lateral-flow disposable strip [23] (e.g., SureStep® technology from Lifescan Inc., Wayne, PA, USA [46]). The test strip comprises a hydrophilic antistrophic membrane with 30–40 μm pores impregnated with a reagent for glucose detection. A blood sample is absorbed by the absorbent pad, and then red blood cells (RBC) are filtered through bigger pores and subsequently, through smaller pores. Finally, the RBCs are trapped at the membrane. Plasma passes through the membrane to react with a reagent to form a color dye for photometric-reflectance measurement. The membrane induces capillary action without external pressure [23]. 

**Figure 3:**
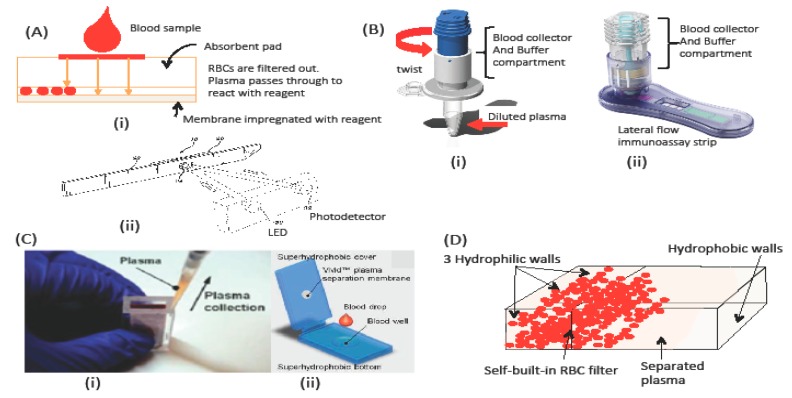
(**A**) Test strip from SureStep® technology. (**B**) FABPulous device designed for heart-type fatty acid binding protein for diagnosis of acute coronary problem. Courtesy of FABpulous, image adapted from www.FAbpoulos.com. (**C**) Plasma collector by Liu et al. group, from [49,50]. and (**D**) In-lab self-built Red Blood Cells (RBC) filter adapted from Reference [53].

Figure 3A (i) and (ii) illustrates the mechanism used in the strip, and the position of the light emitting diode (LED) and photo detector, which use the SureStep® from Lifescan Inc., United States meter to determine the glucose content. In contrast, the FABPulous device detects heart-type fatty acid binding protein for the diagnosis of acute coronary problems. This device uses a manual and disposable membrane-based blood separation platform that relies on a buffer solution to produce a diluted plasma [47]. Figure 3B (i) shows the test platform, which comprises a blood collector and a plasma chamber (white and blue respectively). The filter allows blood plasma to pass through, leaving the blood cells in the chamber. The dilution factor must be adjusted according to the sample volume. This platform integrates directly with a lateral-flow strip for diagnosis, as shown in Figure 3B (ii). This technology requires manual pumping to generate the pressure required for separation. The vertical position of the membrane in the chamber may cause clogging, and strong pressure may result in blood hemolysis [48].

To overcome these issues, Li’s group demonstrated an external-power-free horizontal membrane [49]. They used a pump-free membrane-based filtration method [Figure 3C (i)], with a 100 μm porous membrane installed horizontally in the sample chamber [49]. To reduce clogging at the membrane and to increase plasma production, they use the gravitational sedimentation method, wherein the blood sample is allowed to sediment for several minutes before plasma is collected via pipette. Sedimentation velocity for RBCs range from 0.27 to 3.8 μm/s depending on the patient’s gender, health, and pathologic status [45]. Liu et al. [50] combined this method with cross-flow filtration or tangential-flow filtration, which allows the solution to pass tangentially along the filter or membrane surface. In tangential filtration, smaller particles pass through the filter, whereas larger particles do not. The collected plasma is then amplified in the microfluidic chip. 

In addition to this work, Liu et al. demonstrated another plasma collector that uses the sediment concept in the form of a super-hydrophobic membrane surface [50]. Figure 3C (ii) shows the plasma collector design. In this design, the blood sample is sandwiched between two super-hydrophobic surfaces. By using the sedimentation method, the RBCs flow to the bottom part, leaving plasma at the top, which is then, collected using a pipette. The plasma separator demonstrates a high plasma yield (>70%). The device will soon be integrated with a diagnostic device to make a POC diagnostic system. Although several other works also integrate microfluidics and membrane filters [48,51,52], these devices have only been demonstrated in the laboratory and have yet to be commercialized. 

The commercialized technology discussed thus far remains limited in POC applications because they still require a sample transfer, concomitant expert handling, and separate readout system. Recently, Maria et al. [53] demonstrated a self-built blood filter in a microchannel that comes with mixed hydrophilic-hydrophobic regions in a PDMS-based device. The device performs blood plasma separation for glucose detection. The hydrophilic surface is prepared by exposing the PDMS surface to oxygen plasma. Figure 3D shows a schematic of the device with mixed hydrophilic-hydrophobic regions (vertically divided). When whole blood is inserted into the inlet, the capillary force drags the sample through the hydrophilic channel. In the lower hydrophobic region, the large contact angles decrease the capillary flow. This hydrophobic region thus becomes a flow barrier and causes the accumulation of RBCs. This is also due to the nature of RBCs, which are slow compared with plasma. Although the barrier stops RBCs, plasma flows past the barrier (i.e., filter) and separates from the RBCs. This device recovers 22.5% of the plasma from pure blood. However, this device has only been demonstrated at the lab-scale and is not yet commercialized.

#### 3.1.2. Membrane-based blood-plasma separation 

To date, no commercially available portable stand-alone microfluidics-based devices are available for blood-plasma separation. As discussed in the previous section, the commercialized devices available today mainly use membrane for blood-plasma separation. Exploring the separation process by using membrane can generate new ideas for developing portable IDA device. 

**Figure 4:**
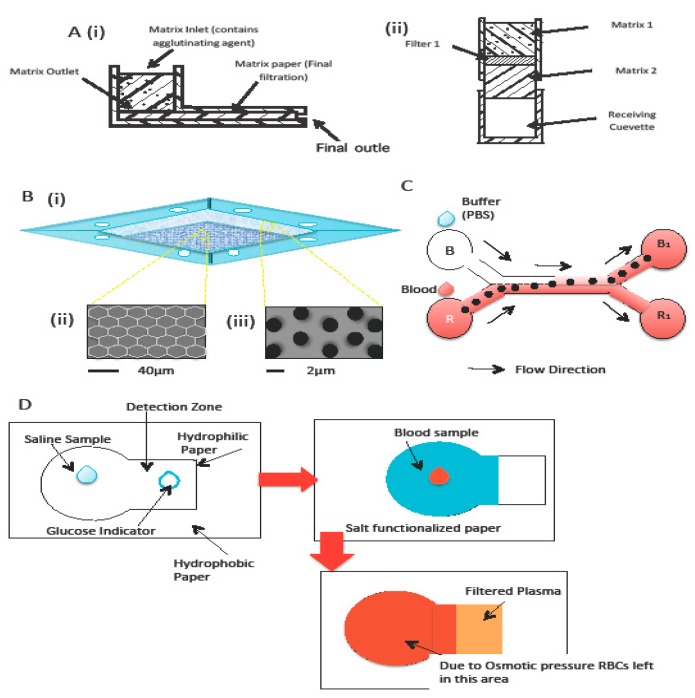
(**A**) Filter that uses matrix of a hydrophilic sintered porous material with different designs taken from Ref. [54]. Panel (**B**) (i) shows a 3D parylene-filter design by Liu et al. Images are modified from [55], where panel (**B**) (ii) shows 40 µm micropores that prevent clogging and panel (**B**) (iii) shows 2 micropores for plasma filtration. (**C**) H-filter paper-based device used by Kar et al. Image are modified from [56]. (**D**) Salt-functionalized paper used to separate plasma from blood by Nilghaz and Shen. Images are modified from [57].

The membrane-based separation method was first introduced by Aunet et al., who designed a plasma separation method that uses a matrix of hydrophilic sintered porous material [54]. Figure 4A (i) shows how the plasma separation process works with this matrix. The design basically comprises an inlet and outlet, with the matrix placed between the two. When whole blood is injected into the inlet, the coagulating agent in the matrix coagulates the blood, thereby trapping the RBCs and, yielding filtered plasma at the outlet. The matrix is made from hydrophilic sintered porous material and acts as a filter. The sintered material can be made from glass, steel, ceramic, or plastic. Aunet et al. used polyethylene and the pore size in the material ranged from 10 to 70 µm. Another design was demonstrated wherein two matrices were sandwiched around a filter to enhance the filtration process. The filtered plasma flows directly to the receiving cuvette [Figure 4A (ii)]. The device was tested using 40 microliters of whole blood, which produced 10 microliters of clear plasma (99% free of hemoglobin) within 2 min.

Following the idea of a porous membrane, Liu et al. demonstrated a method to separate plasma from whole blood using a 3D filter [55]. The 3D filter was prepared by using a parylene membrane, which contains 2 different size micropores (Figure 4B). One type of micropore is 1.64 ± 0.08 µm in size and serves to filter the plasma, and the other type is 39.06 ± 0.39 µm in size and serves as a by-pass channel to prevent clogging. The design used in the experiment was 2 µm for the small micropores [see Figure 4B (ii)] and 40 µm for the large micropores [see Figure 4B (iii)]. This 3D model was also demonstrated using undiluted blood. The device gives a high yield of 42% (volume of separated plasma with respect to the volume of whole blood) at a high throughput (2 µL/5 min).

Instead of using a microfilter, Osborn’s group [58] from the University of Washington developed a H-filter based on 2-Dimensional Paper Networks (2DPNs) wherein a pump forces fluid through a filter and the separation of small particles from complex fluid samples is analyzed. Kar et al. [56] improved this 2D paper-based design by exploiting the law of capillary-driven blood blood transport, as shown in Figure 4C. The system is designed to be a portable device for blood diagnostics in places wherein resources are limited. It uses a Whatman Grade 4 type of filter paper and basically focuses on blood separation (plasma and RBCs). During the process, 50 µL of blood was stored in reservoir B1 and 50 µL of phosphate-buffered saline (PBS) solution (pH~7.4) was stored in reservoir R. Due to the flow trajectory, lighter molecules diffuse into the PBS stream after suspension within the bloodstream. After 200 s, more red stains appear at reservoir R1 than at reservoir B1. Filter-paper-based separation was adapted by Nilghaz and Shen [57], who used a salt-functionalized Whatman Grade 4 filter paper to separate plasma from blood, as shown in Figure 4D. When a whole blood sample was deposited on the salt-functionalized paper, the salt dissolves and creates a hypertonic environment that forces the RBCs to crenate and agglutinate onto the paper (Figure 4D). Plasma could continue flowing forward and be collected at the end of the process. Figure 4D shows the process of blood plasma separation using the salt-functionalized Whatman Grade 4 filter paper. 

Another possible separation technique uses a polycaprolactone-filled glass microfiber membrane [59].

#### 3.1.3. Buffer or No Buffer for Separation?

Because of its high viscosity, blood commonly forms aggregates (clots) in the process of blood plasma separation [39,60,61]. Although many attempts have been made to separate plasma liquid from blood using various macro-scale devices, success has been limited because of the clogging problem [62]. Therefore, a buffer solution is preferred. Hung and Chang mixed a PBS solution with the blood at volume ratio of 1:10 [63] to experimentally demonstrate using rat blood the separation of plasma from whole blood, which is useful for detecting human chorionic gonadotropin (hCG) protein. The main purpose of the PBS solution is to dilute whole blood to a lower viscosity level and prevent clogging. The PBS buffer solution is used to provide a sheath flow to force the RBC to the bottom of the channel. In other work, Yang et al. proposed a separation method using PBS solution in which, defibrinated sheep blood was diluted with 36% initial hematocrit to six different hematocrit levels ranging from 10% to 35% [64]. They achieved a total plasma separation volume of 15%–20%. 

In hydrodynamics-based devices, the fabrication process is facilitated using a buffer solution that helps to generate sheath flow, to force the blood cells to the bottom of the device channel. Paper-based devices are usually simple, low-cost, rapid, and portable. 

#### 3.1.4. Reagent-Deposition and Micro-Mixing

**Figure 5:**
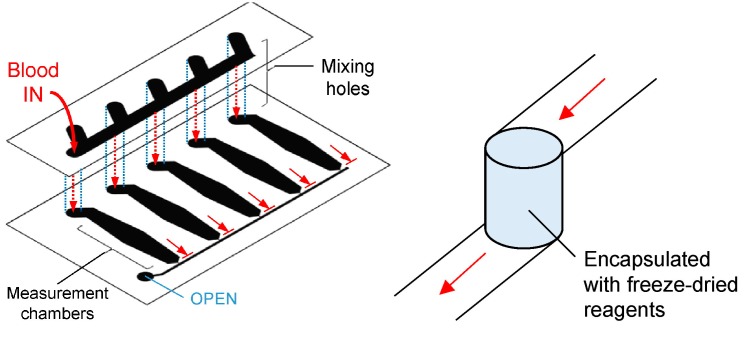
Mixing-hole micro-channels, taken from Reference [67]. For the mixing hole channel, the samples that flow from the upper channel mix with the freeze-dried reagent in the mixing hole and subsequently flow to the lower channel. The device is used to detect hemagglutination.

As opposed to plasma separation, a micro-mixing on-chip device may also be used for diseases requiring special reagents. Sample and reagent mixing in microfluidic devices is a pre-eminent challenge due to the laminar flows that result from low Reynolds number [65]. Lamination [66], mixing-hole method [67] (Figure 5), serpentine structure [25] (Figure 1A (iii)), and helix mixer are some of the micro-mixing designs demonstrated to date. A passive method is preferable for POC to eliminate the need for more equipment. Lee et al. presented an in-depth discussion of micro-mixing in microchannel devices [68]. 

Thus, concepts of sedimentation, cross-flow filtration, capillary action, and as well as mixing design have been used in devices that have been demonstrated as laboratory-scale micro-fluidics chips. Samborski et al. [69] showed the workable all-in-one microfluidic system that integrated sedimentation for blood separation, a plasma-reagent mixing segment, and detection in a single chip. Normally, for sedimentation, a dilution buffer is required to avoid clogging. However, Samborski’s group used a non-diluted, 27 μL samples from a finger prick and recovered 6 μL of plasma from the separation process in less than 10 min. They used a triangular micro-mixing channel for plasma-reagent mixing. Note that capillary-driven flow normally produces multiple bubbles due to spontaneous formation and entrapment. However, in this device, a single bubble forms and helps to increase the mixing rate. The all-in-one chip is motivated by the self-contained, self-powered integrated microfluidic blood analysis system (SIMBAS) developed by Dimov et al. [70], where plasma extraction, multiple-biomarker detection, and suction chambers are integrated into one microfluidic device. The plasma separation segment comprises a round filter trench where plasma is captured via gravity sedimentation to immobilize specific biomarkers on a 15 μm streptavidin bars ceiling and suction chambers for volume assay regulation to ensure that the sample does not overflow into the biomarker detection area. Within 10 min, biotin is detected in less than 5 μL of whole-blood sample. However, this device needs to be pre-conditioned by degassing the chip in a vacuum desiccator (<0.3 atm) for 15 min prior to sample loading, whereas Samborski applied a computer-controlled pump to assist sample flow. 

For rapid measurements, POC diagnostic devices require that reagent be directly integrated into the device. Because microfluidics is designed for measurements of sub-milliliter liquid samples, the integration of consistent and reproducible reagents becomes challenging. Hitbleck and Delamarche [71] presented an in-depth review of the challenges of reagent deposition in microfluidic devices. In addition to the amount of reagent deposited in the device, other factors such as cross contamination, temperature, and precise localization need to be taken into account. Three reagent states are possible in a microfluidic device: dried, liquid, and surface-immobilized state. Numerous technologies have been applied to deposit reagents into these devices such as pipetting (manual or robotic), inkjet or screen printing, and carrier beads [71]. 

Most commercialized lateral-flow strip tests, such as pregnancy-test strips, apply the spray-coating method for reagent deposition [71,72]. Inkjet printers are used to print spots or dots reagent onto the surface [73,74]. Inkjet printing is not a cutting-edge technology in microfluidics or biosensors; this method has already been demonstrated numerous times, especially in paper-based microfluidic devices. Inkjet printing can be used for printing substrates, patterning channels, and depositing assay reagents [75,76,77,78,79]. Inkjet printing involves two main modes of operation: continuous mode and drop-on-demand mode (Liu et al. discuss these methods in depth [80]). However, the drawbacks of this method include exposing the “ink” to heat-pulses, which may affect the biomolecules and also create high-shear forces that may damage proteins during the process [75]. As the storage temperature cannot be controlled, the heat might destroy the functionality of the reagent. Thus, dried reagents are favored over liquid reagents because the former are more stable, especially for applications in developing countries. In addition, reagents dried *in situ* can facilitate device automation because device geometry and reagent location within the device can be designed to automate multistep processes [81].

Conventionally, dried reagents are stored in conjugated pads separated from the microfluidics device and would requires solvent removal after integration into the device [71]. Steven et al. reported an on-card reagent that uses a laminated card with an anhydrous labeling reagent and a laser-cut porous membrane for the malaria antigen *Plasmodium falciparum*histidine-rich protein II [82]. The on-card reagent preparation takes 9 min. The sample is first injected into the card and then the card is clamped in the pump-interface manifold. The reagent is then rehydrated and pushed through the membrane using an instrument script comprising syringe pumps controlled by software. 

However, the reagent is not pre-stored on the chip in this method. Therefore additional materials are required, which increases the manufacturing cost. Brivio et al. [83], via their OPTPLABCARD and SMART-BioMEMS projects, demonstrated a freeze-dried reagent stored on-chip to amplify the polymerase chain reaction (PCR). To store the freeze-dried reagent, a premixed solution of PCR reagent is injected into the reaction chambers and the chips are stored overnight at −80 °C prior to transferring to a freeze drier. Another method to integrate and release dried reagent into a microfluidic device, is called “reagent integration” [84]. This method, allows the user to control the amount of reconstituted reagent to be integrated into the device for a pre-determined sample volume. This reagent is then injected into the reagent integrator by an inkjet deposition process. For testing, the sample is manually pipetted into a loading pad and capillary action will then drag the sample to the capillary pump at the end of the chip. This method may be used in an advanced layout wherein the reagent integrator may be built in parallel for multiple reagents on one chip. 

## 4. Outlook and Challenges of Portable Microfluidics System for Iron Deficiency Anemia Detection

This review focuses primarily on the potential of developing on-chip microfluidic systems for IDA detection. Table 2 summarizes the technology discussed herein. The disadvantages of each invention can be improved upon in future IDA-POC devices. Developing an all-in-one micro-device is a challenge because all stages require an appropriate calculation and careful design, from sample collection and volume determination to blood-plasma separation, reagent storage, sample mixing, and signal readout system. Although such devices are already commercialized for specific diseases, the inability of these devices to meet stringent detection criteria can result in dead-end application. 

The first consideration when developing a diagnostic device is to recognize the specific target at extremely low concentration. For example, for IDA detection, the cut-off level and reference range of biomarkers differ based on the age population, wherein the serum ferritin concentration for children (1 to 5 years of age) and adults is 12 ng/mL and 15 ng/mL, respectively [85]. Although a few studies demonstrated the use of such devices for blood-plasma separation, the search continues for an effective and efficient blood separation device that works in particular for low-volume, undiluted blood. This search is hindered by several aspects, such as a lack of an in-depth understanding of cell and flow behavior for design optimization, choices of material for microfluidic devices, and excessive manufacturing costs [39]. On-chip reagent storage using dried reagent is an attractive approach because this type of reagent is more resistant to damage compared to a liquid reagent. A proper selection of reagent deposition method and the amount of reagent to be deposited need to be considered. Finally, to facilitate use, the signal and readout system should also be integrated onto the microfluidic device. 

**Figure 6:**
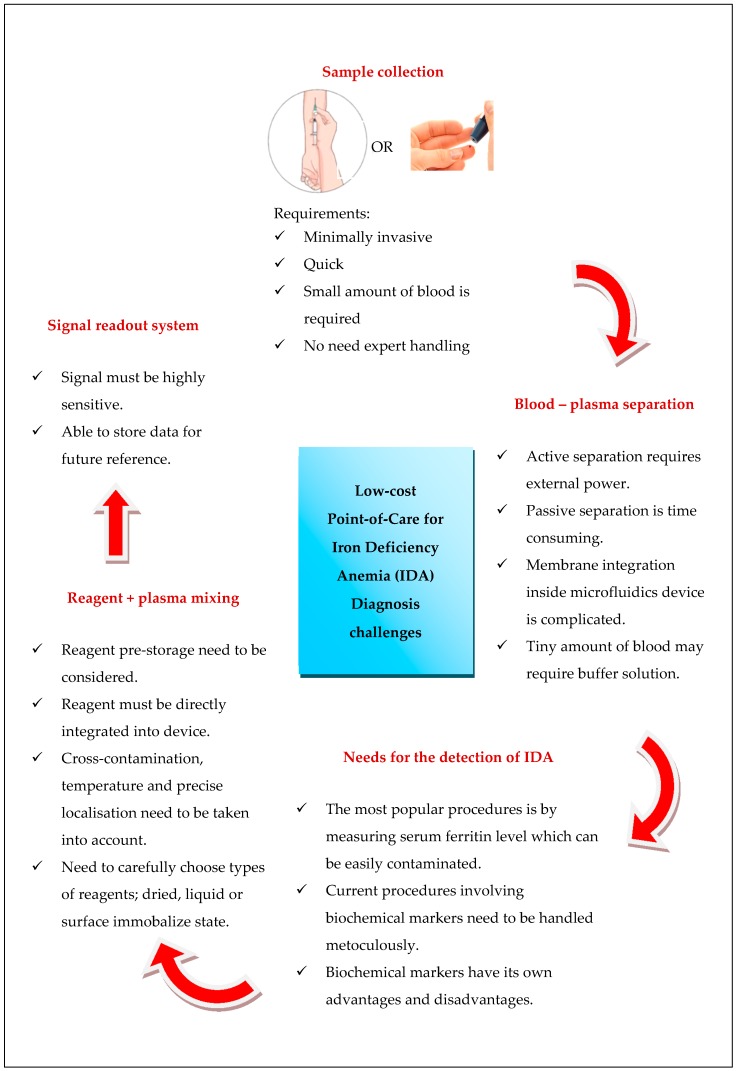
Challenges for developing an all-in-one portable iron deficiency anemia (IDA).

Figure 6 summarizes the current challenges to developing a portable IDA system. All these points are crucial for designing microfluidic devices for portable IDA-POC detection in a low-resource environment.

## Figures and Tables

**Table 1:** Biochemical markers and their respective concentrations for iron deficiency anemia (IDA) screening [3].

Biochemical Marker Serum Ferritin	Normal N	Iron Depletion D	Iron Deficiency Without Anemia N	Iron Deficiency Anemia D
(mcg/dL) (mcg/L)	100 ± 60	<20 (200)	≤10 (100)	<10 (100)
Serum iron	(1000 ± 600)			
	N	N	D	D
(mcg/dL) (mcg/L)	115 ± 50	<115 (20.6)	<60 (10.7)	<40 (7.2)
Total iron-binding	(20.6 ± 9.0)			
	N	N	N/I	I
Capacity	330 ± 30	360 to 390	390 to 410	≥410 (73.4)
	(59.0 ± 5.4)	(64.6 to 69.8)	(69.8 to 73.4)	
Transferrin	N	N	D	D
Saturation (%)	35 ± 15	<30	<20	<10
Serum transferring	N	I	I	I
Receptor (nmol/L)	<35	<35	≥35	≥35
Zinc	N	N	I	I
Protoporphyrin/He	<40	<40	≥40	≥70
me (µmol/mol)				

N = normal, I = increased, D = decreased
